# Exploring the Relationship Between Dietary Inflammatory Index, Eating Disorder, and Body Composition Among University Students in the UAE: A Cross‐Sectional Study

**DOI:** 10.1002/hsr2.73357

**Published:** 2026-10-08

**Authors:** Leila Cheikh Ismail, MoezAlIslam E. Faris, Dana N. Abdelrahim, Maysm Shapsogh, Amira Imad Al Mast, Hind Majdi Abuyounis, Sara Atheel Altekreeti, Lily Stojanovska, Ayesha S. Al Dhaheri

**Affiliations:** ^1^ Department of Clinical Nutrition and Dietetics, College of Health Sciences University of Sharjah Sharjah UAE; ^2^ Nuffield Department of Women's & Reproductive Health University of Oxford Oxford UK; ^3^ Department of Clinical Nutrition and Dietetics, Faculty of Allied Medical Sciences Applied Science Private University Amman Jordan; ^4^ Research, Institute for Medical and Health Sciences University of Sharjah Sharjah UAE; ^5^ Department of Nutrition and Health, College of Medicine and Health Sciences United Arab Emirates University Al Ain UAE; ^6^ Institute for Health and Sport Victoria University Melbourne Australia

**Keywords:** body composition, body mass index, dietary inflammatory index, eating disorder

## Abstract

**Background and Aims:**

Dietary inflammatory potential and disordered eating behaviors may influence body composition among young adults. This study aimed to examine the associations between the Dietary Inflammatory Index (DII), eating attitudes assessed using the Eating Attitudes Test‐26 (EAT‐26), and body composition among university students in the United Arab Emirates.

**Methods:**

This cross‐sectional study included 911 university students aged 18–30 years, of whom 50.5% were female. Dietary intake was assessed using a validated food frequency questionnaire, and DII scores were calculated using 28 available dietary parameters. Eating attitudes were assessed using the EAT‐26, and anthropometric and body‐composition measurements were obtained using standardized procedures.

**Results:**

Based on BMI, 26.1% of participants were living with overweight and 11.9% were living with obesity. The mean DII score was −0.77, and 75.4% of participants had an anti‐inflammatory dietary profile, the mean E‐DII score was 2.24 (SD 1.07), with 96.8% classified as pro‐inflammatory. Based on EAT‐26 scores, 30.3% were classified as being at major eating‐disorder risk. Compared with the pro‐inflammatory group, participants in the anti‐inflammatory group had significantly lower body fat percentage and fat mass and higher total body water percentage. DII showed a significant but weak negative correlation with total body water percentage only; no other significant DII correlations with body composition were observed. EAT‐26 scores were significantly associated with several body‐composition measures, including body mass index, fat mass, and visceral fat rating. As a sensitivity analysis, EAT‐26 scores also differed significantly by E‐DII group (mean difference = 5.34, 95% CI: 0.65–10.04, *p* = 0.026), with higher scores in the anti‐inflammatory group.

**Conclusion:**

Eating attitudes showed stronger associations with body‐composition measures than dietary inflammatory potential in this population. Hence, promoting healthy eating behaviors and identifying disordered eating risk among university students are important.

## 1 Introduction

The transition from adolescence to young adulthood is a critical developmental period during which major lifestyle changes may influence dietary behaviors and long‐term health outcomes [1]. University students are generally perceived as a relatively healthy population; however, they are vulnerable to health‐risk behaviors and mental health problems [2]. Academic stress and time constraints may contribute to unhealthy eating behaviors among university students, including frequent consumption of energy‐dense and fast foods, inadequate fruit and vegetable intake, irregular eating patterns, and meal skipping [3, 4]. Such dietary behaviors are associated with excess adiposity and an increased risk of overweight and obesity during young adulthood [5]. University entry also represents a major life transition characterized by increased independence and greater control over food choices, making it an important period for establishing dietary habits that may persist into adulthood [1].

The association between diet, inflammation, eating behaviors, and body composition has garnered increasing attention from researchers and healthcare professionals in recent years. This focus is rooted in the understanding that diet can modulate inflammatory responses in the body and, in turn, affect health outcomes [6]. The Dietary Inflammatory Index (DII) is a literature‐derived, population‐based scoring algorithm developed to quantify the inflammatory potential of an individual's diet [7]. The original DII was developed using 45 dietary parameters, including nutrients, foods, and bioactive compounds [7]. In the present study, the DII was calculated using 28 dietary parameters available from the dietary assessment data.

Negative DII scores indicate a more anti‐inflammatory dietary profile, whereas positive scores indicate a more pro‐inflammatory profile. Higher DII scores have been associated with increased risks of several chronic disease outcomes, although the strength of evidence varies across outcomes and does not establish causality [8]. Conversely, greater adherence to an anti‐inflammatory dietary pattern may be associated with better quality of life [9]. Previous observational evidence suggests that lower DII scores are associated with lower body‐fat percentage, whereas higher scores are associated with greater adiposity [10, 11]. Consistently, recent meta‐analytic evidence indicates that higher DII scores are associated with increased odds of abdominal obesity and metabolic syndrome [12].

Concurrently, disordered eating and the risk of eating disorders, including anorexia nervosa, bulimia nervosa, and binge‐eating disorder, represent important physical and psychological health concerns among university students [13, 14]. Dietary attitudes and certain food items may influence inflammation and body composition. Therefore, a cross‐sectional study among adults with abdominal obesity found that greater adherence to a Western dietary pattern was associated with higher BMI and fat mass, whereas adherence to a traditional dietary pattern was associated with lower DII scores [15]. Similarly, the CUME study found that participants in the highest E‐DII quartile consumed more red and ultra‐processed meats, fats and oils, sugar‐sweetened beverages, sugars, sweets, and total energy and had a higher prevalence of overweight and obesity [16]. Prospective findings from the SUN cohort further showed that participants in the highest DII quartile had a 32% greater risk of developing overweight or obesity than those in the lowest quartile (HR: 1.32; 95% CI: 1.08–1.60) [17].

There is a scarcity of studies that shed light on the intricate relationship between dietary choices, inflammatory potential, eating disorders, and body composition among university students. Therefore, we aim to explore whether the inflammatory potential of one's diet could be linked to the development of eating disorders and whether it influences body composition. Understanding these relationships is of paramount importance, as it can guide the development of targeted interventions and educational programs aimed at promoting healthy eating behaviors, preventing eating disorders, contributing to the promotion of holistic well‐being, and the formulation of evidence‐based strategies to support the health of university students during this pivotal stage of life.

## 2 Methods

### 2.1 Study Design and Subjects

In this quantitative cross‐sectional study, 911 healthy students aged 18–30 were recruited from the University of Sharjah, UAE. The target population was students registered at the University of Sharjah during the academic year of 2018/2019. Students with a history of bone disease, metabolic or endocrine diseases, and hormone contraceptive therapy, which could affect bone mass, were excluded. Additionally, subjects with major acute or chronic conditions who had made significant lifestyle changes in the preceding months that could have affected their body weight were excluded. Participants were recruited based on non‐probability quota sampling from the university's four campuses. The sample size was calculated using a sample size calculator, with a margin of error of 5%, a confidence level of 95%, and a response distribution of 50%, yielding 384 participants. An additional 20% (attrition rate) was added, yielding approximately 461 participants. Researchers approached students and invited them to participate in the study after verbally explaining the study's significance, objectives, and protocol. Those who agreed to participate in the study were provided with an information sheet and a consent form. To minimize potential sources of measurement and reporting bias, standardized, calibrated equipment was used for all anthropometric assessments, previously validated instruments were used for dietary and eating‐attitude assessment, and participant anonymity was maintained throughout data collection to reduce social‐desirability bias in self‐reported responses.

### 2.2 Ethical Considerations

This study was conducted in accordance with the principles of the Declaration of Helsinki. Ethical approval for all procedures involving human participants was obtained from the Research Ethics Committee at the University of Sharjah (Reference No. REC‐20‐05‐26‐02‐S). Written informed consent was obtained from all participants prior to their participation in the study.

### 2.3 Anthropometric Assessment and Body Composition

Body weight (BW), fat mass (FM), fat‐free mass (FFM), bone mass (BM), muscle mass (MM), body fat percentage (BF%), total body water (TBW), basal metabolic rate (BMR), and visceral fat rating (VFR) were measured twice (without shoes and in lightweight clothes) to the nearest 0.11 kg using a body composition analyser (TANITA BC‐420MA, Tanita Corp., Tokyo, Japan). A SECA 769 ‐ Digital column scale (Seca Deutschland, Hamburg, Germany) was used to measure height. Each participant was asked to stand erect with their back, buttocks, and heels in continuous contact with the vertical height rod of the stadiometer, and their head orientated in the Frankfurt plane. The horizontal headpiece was then placed on top of the participant's head to measure their height. Height was measured twice, without shoes, to the nearest 0.5 cm. The average of the two values for each measurement was used in the analysis. VFR was recorded as reported by the Tanita BC‐420MA analyser and was analysed in its original scale.

### 2.4 Survey Questionnaires

A validated self‐reported questionnaire consisting of three sections was administered to the participants. The first part included sociodemographic questions inquiring about gender, age, nationality, marital status, living place, smoking status, monthly income, college, and year level of study. The second part included a food frequency questionnaire (FFQ) which was comprised of 65 food items and the respondents had to place a tick based on their dietary intake over the previous year, then each food item was analysed using ESHA software. The last section included the eating attitudes test (EAT‐26).

### 2.5 Dietary Measurements and DII Calculation

Dietary assessment was carried out by a self‐reported, validated, and reliable 65‐item quantitative FFQ adapted from El Mesmoudi et al. 2022 that a trained nutritionist administered to assess the average daily intake for last year [18]. The FFQ consisted of a list of foods with standard serving sizes and participants were asked to report their usual intake for each food item consumed. The FFQ contained nine response options: (1) Never; (2) 1–3 times per month; (3) once per week (4) 2–4 times per week (5) 5–6 times per week; (6) Once per day; (7) 2–3 times per day; (8) 4–5 times per day; and (9) more than 6 times per day. Household measures were used to convert portion sizes of the consumed foods into grams, and an estimated average daily intake of food parameters was calculated from the FFQ using Food Processor Nutrition Analysis Software‐ESHA (version 10.4; ESHA Research, Salem, OR, USA).

For these participants, total energy intake and all 28 associated food parameters were corrected using a proportional scaling approach: each participant's reported intake of every food parameter was scaled by the same factor used to bring their total energy intake into a plausible range, thereby preserving each participant's reported macronutrient distribution while removing the implausible absolute magnitude.

FFQ‐derived dietary data were used to calculate DII scores for all participants. The dietary data were linked to the regionally representative world database that included food consumption from 11 populations around the world and provided a robust estimate of a mean and standard deviation for each parameter [23]. This yielded a standardized z‐score, the “standard global mean”, for each parameter, calculated as the difference between the individual's intake and the global mean, divided by the standard deviation. Because raw intake values are typically right‐skewed, each z‐score was then converted to a percentile rank and rescaled to a symmetric distribution by doubling the percentile and subtracting 1, producing a value bounded between ‐1 and +1 for each parameter. This rescaled score was weighted by the parameter's literature‐derived inflammatory effect score to yield a parameter‐specific inflammatory contribution, and the parameter‐specific contributions were summed across all 28 parameters to produce each participant's overall DII score [23]. A total of 28 food parameters were available from the FFQ, and were used to calculate DII [namely: total calories (Kcal), protein (g), carbohydrate (g), fiber (g), fat (g), saturated fat (g), MUFA (g), PUFA (g), trans fat (g), cholesterol (mg), omega 3 (g), omega 6 (g), vitamin A (IU), beta‐carotene (mcg), Thiamine (mg), Riboflavin (mg), Niacin (mg), vitamin B6 (mg), vitamin B12 (mcg), vitamin C (mg), vitamin D (IU), vitamin E (mg), folic acid (mcg), iron (mg), magnesium (mg), selenium (mcg), zinc (mg) and caffeine (mg)]. A positive overall score reflects greater pro‐inflammatory potential of the diet, while a negative score reflects greater anti‐inflammatory potential. Participants were classified into pro‐inflammatory or anti‐inflammatory dietary groups based on the sign of their overall DII score [23].

To address the concern that the crude DII may be masked by total caloric volume, an Energy‐Adjusted DII (E‐DII) was additionally calculated using the nutrient density method: each of the 28 food parameters was expressed as an amount per 1,000 kcal of total energy intake prior to the z‐score, percentile, and effect‐score‐weighting steps described above, applied to the same global reference database. DII was retained as the primary exposure variable for the main comparative analyses (Tables 3, 4, and 5), as the E‐DII classification in this sample was severely imbalanced (3.2% anti‐inflammatory vs. 96.8% pro‐inflammatory), which was determined to reflect this population's dietary pattern relative to the DII's reference database rather than residual data error, and which yields unstable estimates for group comparisons given the resulting small subgroup size. E‐DII is reported descriptively (Table 2), as a parallel correlation analysis (Table 6), and as a sensitivity analysis for the EAT‐26 comparison (Supporting Information S1: Table S1).

### 2.6 Eating Disorder Attitudes

Eating disorder attitudes were assessed using the Eating Attitudes Test (EAT‐26) by Garner et al. [24]. EAT‐26 is a 26‐item self‐administered questionnaire, with one reverse‐scored item (item 26). The answers are scored from 0 to 3 as follows: always = 3, usually = 2, often = 1, and sometimes/rarely/never = 0, with the last question receiving a reversed score. The total score ranges from 0 to 78 and is categorized into two groups i‐e ≥ 20 indicates major eating disorder risk and < 20 indicates minor eating disorder risk [25]. Three sub‐scale scores are calculated, namely (i) Dieting, (ii) Bulimia and Food Preoccupation, and (iii) Oral Control. The EAT‐26 questionnaire is not used to diagnose eating disorders but only as a screening tool that can detect possible cases of eating disorders, and those with an indicative score should seek further evaluation from health experts. In the present study, the Cronbach α for the EAT‐26 was calculated at 0.79.

### 2.7 Statistical Analysis

Categorical variables were reported as frequencies and percentages, while continuous variables were summarized as mean and standard deviation (SD). BMI was calculated as weight over height squared (kg/m^2^) and was classified into four categories i.e underweight (< 18.5 kg/m^2^), normal (18.5–24.9 kg/m^2^), overweight (25.0–29.9 kg/m^2^), and obese (> 30.0 kg/m^2^) following WHO criteria [26]. The primary, pre‐specified analyses were: (i) comparison of body‐composition measures and EAT‐26 scores between DII groups (Table 3), and (ii) the association of DII and EAT‐26 scores with body‐composition measures (Table 6), consistent with the study's stated aim. The comparison of individual nutrient intakes between DII groups (Table 4) and the subgroup comparison of BMI by EAT‐26 risk category within each BMI classification (Table 5) were exploratory, post‐hoc analyses conducted to further characterize the primary findings and were not corrected for multiple comparisons; results from these analyses should be interpreted as hypothesis‐generating. Independent‐samples t tests were used to compare continuous variables between the pro‐inflammatory and anti‐inflammatory dietary groups (Tables 3, and 4). Because Levene's test indicated unequal variances for most comparisons in the corrected dataset, and given the imbalanced group sizes, Welch's t‐test (not assuming equal variances) is reported throughout as the primary test, consistent with recommended practice under these conditions. For all group comparisons, the mean difference and its 95% confidence interval (CI) are reported alongside Cohen's d as a standardized measure of effect size. Associations of DII and EAT‐26 scores with body‐composition measures were assessed using Pearson's correlation coefficient (r); 95% CIs for correlation coefficients were reported. All tests were two‐sided, and *p* < 0.05 was considered statistically significant. Statistical analyses were performed using IBM SPSS Statistics version 30.0 (SPSS Inc., Chicago, IL, USA).

## 3 Results

Table 1 presents the sociodemographic characteristics of the students. The percentage of male and female students was almost the same (49.5% vs. 50.5%). The age range of students was 18–30 years, but the majority (76.5%) were under 21 years. About 95.7% of students were single, while the majority (45.2%) belong to the Arab non‐GCC (Gulf Countries Council) region. Most of the students (65.2%) were living with families, while 32.2% were living in dormitories. About 23.9% of students were smokers, while 73.5% of the students had monthly family income below 5000 United Arab Emirates Dirham (AED). Almost half of the students (49.7%) were enrolled in medical and health sciences courses followed by applied physics (37.3%) and humanities (13%). Majority of the students were in the first and third year of their study. Further, the nutritional status of the students was categorized based on the BMI value. Approximately 55.3% of students reported normal weight, while 26.1% and 11.9% were reported as overweight and obese, respectively.

**Table 1:** Sociodemographic characteristics of the students (*n* = 911).

Variable		Frequency (%)
Gender	Male	451 (49.5)
	Female	460 (50.5)
Age group	18–21 years	697 (76.5)
	22–30 years	214 (23.1)
Nationality	UAE Citizen	160 (17.6)
	Arab GCC	259 (28.4)
	Arab non‐GCC	412 (45.2)
	Non‐Arab	80 (8.8)
Marital status	Single	872 (95.7)
	Married	22 (2.4)
	Divorced/widowed	17 (1.9)
Living place	Dorms	293 (32.2)
	Outside with family	594 (65.2)
	Outside with friends	24 (2.6)
Smoking	Yes	218 (23.9)
	No	693 (76.1)
Monthly Income	Less than 5000 AED	670 (73.5)
	5,000 AED and above	241 (26.4)
College	Humanities	118 (13.0)
	Applied Physics	340 (37.3)
	Medical and Health sciences	453 (49.7)
Educational level	Junior 1st, 2nd, 3rd year	399 (43.8)
	Senior 4th, 5th, 6th year	512 (56.2)
BMI Categories	Underweight	61 (6.7)
	Normal weight	504 (55.3)
	Overweight	238 (26.1)
	Obese	108 (11.9)

Following identification and correction of implausible dietary intake data (see Statistical analysis), the mean DII score was −0.77 (SD 1.46) and the mean E‐DII score was 2.24 (SD 1.07); the mean EAT‐26 score was 15.07 (SD 12.70). Based on the DII score, students were categorized into anti‐inflammatory (75.4%) and pro‐inflammatory (24.6%) groups; based on the E‐DII score, 3.2% were anti‐inflammatory and 96.8% were pro‐inflammatory. Based on the EAT score, 30.3% of the students were at risk of major eating disorders and 69.7% were at risk of minor eating disorders (Table 2).

**Table 2:** Dietary inflammatory index and eating attitudes score of the students (*n* = 911).

Variables		Mean ± SD
Dietary Inflammatory Index (DII) Score		−0.77 ± 1.46
Energy Adjusted Dietary Inflammatory Index (E‐DII) Score		2.24 ± 1.07
Eating Attitude Test Score		15.07 ± 12.70
		**Frequency (%)**
DII score categories	Anti‐inflammatory diet (Negative values of DII)	687 (75.4)
	Pro‐inflammatory diet (Positive values of DII)	224 (24.6)
E‐DII score categories	Anti‐inflammatory	29 (3.2)
	Pro‐inflammatory	882 (96.8)
Eating Attitude Test	Major eating disorder (≥ 20) riskpositive/high‐risk screen	276 (30.3)
	Minor eating disorder (< 20) risknegative/low‐risk screen	635 (69.7)

Table 3 presents the comparison of body composition and EAT‐26 score between the DII anti‐inflammatory and pro‐inflammatory groups, together with the distribution of BMI categories within each group. Body fat percentage, fat mass, and total body water percentage were significantly different between groups (all *p* < 0.05), with the anti‐inflammatory group showing lower body fat percentage and fat mass and higher total body water percentage. No other body‐composition measure, nor EAT‐26 score, differed significantly between DII groups. BMI category distributions were broadly similar between groups. As a sensitivity analysis using the E‐DII grouping (Supplementary Table S1), EAT‐26 score differed significantly between groups (mean difference = 5.34, 95% CI: 0.65–10.04, *p* = 0.026), with the anti‐inflammatory group reporting higher EAT‐26 scores than the pro‐inflammatory group.

**Table 3:** Comparison of body composition and EAT between anti‐inflammatory and pro‐inflammatory groups (*n* = 911).

Variables	Overall mean	Mean (SD)		Mean difference (95% CI)	*p* value	Cohen's d	
		Anti‐inflammatory *N* = 687	Pro‐inflammatory *N* = 224				
Weight (Wt.)	70.06 (18.30)	69.53 (17.90)	71.67 (19.43)	−2.14 (−5.02, 0.75)	0.146	−0.117
Height (Ht.)	168.33 (9.91)	168.18 (9.79)	168.81 (10.28)	−0.63 (−2.17, 0.91)	0.421	−0.064
Fat percent (Fat%)	22.41 (9.64)	21.99 (9.52)	23.69 (9.92)	−1.70 (−3.18, −0.21)	0.025*	−0.177
Fat mass (FM)	16.39 (10.30)	15.99 (10.25)	17.63 (10.37)	−1.65 (−3.21, −0.08)	0.039*	−0.160
Fat free mass (FFM)	53.75 (13.10)	53.65 (12.93)	54.08 (13.64)	−0.43 (−2.47, 1.61)	0.677	−0.033
Muscle mass (MM)	51.01 (12.44)	50.96 (12.18)	51.17 (13.23)	−0.21 (−2.17, 1.75)	0.834	−0.017
Total body water (TBW)	38.06 (9.01)	38.07 (8.97)	38.02 (9.16)	0.05 (−1.33, 1.42)	0.949	0.006
Total body water % (TBW%)	54.91 (6.50)	55.27 (6.40)	53.83 (6.69)	1.44 (0.44, 2.44)	0.005*	0.222
Bone mass (BM)	2.71 (0.61)	2.71 (0.60)	2.72 (0.64)	−0.01 (−0.11, 0.09)	0.828	−0.016
Basal metabolic rate (BMR)	1,652.43 (374.08)	1648.40 (364.53)	1664.79 (402.55)	−16.40 (−75.95, 43.16)	0.589	−0.044
Visceral fat rating (VFR)	3.11 (3.42)	3.03 (3.46)	3.33 (3.29)	−0.30 (−0.81, 0.20)	0.237	−0.088
Body mass index (BMI)	24.51 (5.03)	24.38 (4.96)	24.89 (5.22)	−0.52 (−1.30, 0.27)	0.195	−0.102
Eating Attitude test (EAT) score	15.07 (12.70)	15.42 (12.68)	13.98 (12.75)	1.44 (−0.49, 3.37)	0.142	0.114
	**BMI classification**						
Underweight	61 (6.7)	48 (7.0)	13 (5.8)		0.628	
Normal weight	504 (55.3)	385 (56.0)	119 (53.1)			
Overweight	238 (26.1)	177 (25.8)	61 (27.2)			
Obese	108 (11.9)	77 (11.2)	31 (13.8)			

*Note:* An independent sample *t*‐test was used to assess the difference. *p < 0.05 is considered significant.

We compared the nutrient intake of the students based on the DII score (anti‐inflammatory vs. pro‐inflammatory) to explore the inflammatory potential of individual nutrients (Table 4). Twenty‐two of the 28 dietary variables examined differed significantly between groups (Welch's *t*‐test, *p* < 0.05), including energy, protein, carbohydrate, fiber, fat, saturated fat, MUFA, PUFA, cholesterol, omega‐6, vitamin A, beta‐carotene, thiamine, niacin, vitamin B6, vitamin B12, vitamin C, vitamin D, folic acid, iron, magnesium, and zinc. Trans fat, omega‐3, riboflavin, vitamin E, selenium, and caffeine did not differ significantly between groups.

**Table 4:** Comparison of dietary intake between anti‐inflammatory and pro‐inflammatory groups (*n* = 911).

Variable	Overall mean (SD)	Mean (SD)		Mean Difference (95% CI)	*p* value	Cohen's d
		Anti‐inflammatory *N* = 687	Pro‐inflammatory *N* = 224			
Energy (Kcal)	2,646.44 (901.46)	2860.74 (843.96)	1989.20 (741.37)	871.54 (755.42, 987.67)	< 0.001*	1.063
Protein (g)	90.90 (40.54)	98.47 (35.23)	67.70 (46.59)	30.78 (24.10, 37.45)	< 0.001*	0.803
Carbs (g)	346.21 (138.63)	380.37 (132.83)	241.44 (97.61)	138.93 (122.71, 155.16)	< 0.001*	1.110
Fibers (g)	34.61 (15.69)	39.85 (14.15)	18.52 (6.68)	21.32 (19.95, 22.70)	< 0.001*	1.676
Fat (g)	110.62 (63.64)	118.50 (67.86)	86.46 (39.82)	32.04 (24.75, 39.33)	< 0.001*	0.515
Saturated Fats (g)	39.85 (17.41)	42.40 (16.71)	32.04 (17.24)	10.36 (7.77, 12.95)	< 0.001*	0.615
MUFA (g)	27.23 (11.81)	29.45 (11.46)	20.44 (10.20)	9.02 (7.43, 10.61)	< 0.001*	0.807
PUFA (g)	18.68 (8.59)	20.19 (8.55)	14.06 (6.92)	6.13 (5.02, 7.24)	< 0.001*	0.749
Trans fats (g)	1.76 (28.24)	0.52 (0.82)	5.56 (56.86)	−5.03 (−12.52, 2.45)	0.187	−0.179
Cholesterol (mg)	315.49 (301.26)	331.69 (208.67)	265.81 (482.83)	65.88 (0.44, 131.32)	0.049*	0.220
Omega 3 (g)	1.80 (2.66)	1.85 (0.84)	1.66 (5.17)	0.19 (−0.49, 0.87)	0.583	0.071
Omega 6 (g)	16.53 (8.42)	17.88 (8.58)	12.39 (6.32)	5.49 (4.44, 6.54)	< 0.001*	0.679
Vitamin A (IU)	8,975.24 (8,135.03)	10519.97 (8690.43)	4237.62 (2798.52)	6282.34 (5535.29, 7029.40)	< 0.001*	0.818
Beta carotene (mcg)	3,195.53 (2,668.67)	3815.52 (2763.49)	1294.06 (867.96)	2521.46 (2285.30, 2757.62)	< 0.001*	1.034
Thiamine (mg)	2.00 (1.04)	2.21 (1.04)	1.34 (0.74)	0.87 (0.74, 0.99)	< 0.001*	0.892
Riboflavin (mg)	2.79 (4.12)	2.90 (1.71)	2.46 (7.76)	0.44 (−0.59, 1.47)	0.403	0.107
Niacin (mg)	27.85 (13.29)	30.53 (12.75)	19.63 (11.45)	10.90 (9.12, 12.68)	< 0.001*	0.876
Vitamin B6 (mg)	16.03 (21.94)	17.56 (23.77)	11.32 (14.04)	6.23 (3.67, 8.79)	< 0.001*	0.286
Vitamin B12 (mcg)	7.17 (12.78)	7.98 (13.94)	4.69 (7.78)	3.29 (1.83, 4.75)	< 0.001*	0.259
Vitamin C (mg)	158.76 (147.04)	183.13 (155.79)	84.02 (78.26)	99.11 (83.57, 114.65)	< 0.001*	0.704
Vitamin D (IU)	108.04 (130.46)	123.74 (143.05)	59.90 (58.49)	63.84 (50.66, 77.01)	< 0.001*	0.500
Vitamin E (mg)	22.25 (91.51)	20.57 (32.44)	27.39 (175.78)	−6.82 (−30.09, 16.45)	00.564	−0.075
Folic Acid (mcg)	258.86 (518.49)	288.76 (572.22)	167.14 (280.30)	121.62 (65.16, 178.09)	< 0.001*	0.236
Iron (mg)	267.81 (452.24)	299.34 (497.62)	171.09 (245.75)	128.25 (78.97, 177.52)	< 0.001*	0.286
Magnesium (mg)	252.79 (205.84)	288.68 (216.96)	142.71 (109.31)	145.97 (124.30, 167.64)	< 0.001*	0.744
Selenium (mcg)	196.50 (460.95)	194.52 (169.69)	202.56 (882.29)	−8.03 (−124.89, 108.82)	.892	−0.017
Zinc (mg)	41.81 (55.84)	45.52 (60.43)	30.45 (36.30)	15.08 (8.50, 21.65)	< 0.001*	0.272
Caffeine (mg)	48.13 (80.54)	48.43 (80.34)	47.22 (81.31)	1.21 (−11.05, 13.48)	0.846	0.015

*Note:* An independent sample *t*‐test was used to assess the difference. *p < 0.05 is considered significant.

Following the BMI classification, the nutritional status of the students was categorized into underweight, normal weight, overweight, and obese, and compared by EAT‐26 risk category within each BMI stratum (Table 5). The findings suggested that BMI differed significantly between major and minor eating‐disorder risk groups only within the normal‐weight stratum (*p* = 0.005), where students at minor risk had slightly higher BMI than those at major risk; no significant differences were observed within the underweight, overweight, or obese strata.

**Table 5:** Nutritional status of the students based on the EAT score^$^ (*n* = 911).

BMI Classification	Total population	Mean ± SD		Model summary, *p* value*	Cramér's V
		Major eating disorder risk (*n* = 276)	Minor eating disorder risk (*n* = 635)		
Underweight	61 (6.7)	16 (26.2%)	45 (73.8%)	χ^2^ (3) = 18.938, *p* < 0.001	0.144
Normal weight	504 (55.3)	131 (26.0%)	373 (74.0%)		
Overweight	238 (26.1)	79 (33.2%)	159 (66.8%)		
Obese	108 (11.9)	50 (46.3%)	58 (53.7%)		

*Note:* $ Chi‐square test was used to assess the difference. *p < 0.05 is considered significant.

Table 6 presents the correlation of DII, E‐DII, and EAT‐26 with body composition. DII was significantly, though weakly, negatively correlated with total body water percentage (*r* = −0.080, *p* = 0.015) only; no other DII correlations reached significance. E‐DII showed no statistically significant correlations with anybody‐composition measure, though the correlation with BMI approached significance (*r* = −0.064, *p* = 0.054). In contrast, EAT‐26 score was significantly positively correlated with BMI, fat mass, fat‐free mass, muscle mass, total body water, bone mass, BMR, VFR, and weight (all *p* < 0.05, several *p* < 0.001), and significantly negatively correlated with total body water percentage (*p* < 0.001); no significant correlation was observed between EAT‐26 and height.

**Table 6:** Correlation of DII and eating attitude test with body composition (*n* = 911)^$^.

	DII		E‐DII		EAT	
	Correlation	(95% CI) *p* value	Correlation	(95% CI) *p* value	Correlation	(95% CI) *p* value
BMI	0.038	(−0.03, 0.11) 0.254	−**0.064**	**(−0.14, 0.01) 0.054**	0.155**	**(0.09, 0.23)** < **0.001**
FM	0.054	(−0.01, 0.13) 0.104	−0.057	(−0.13, 0.02) 0.086	0.148**	**(0.08, 0.23)** < **0.001**
FFM	−0.001	(−0.07, 0.07) 0.984	−0.041	(−0.11, 0.03) 0.221	0.079*	**(0.01, 0.14) 0.017**
MM	−0.004	(−0.07, 0.07) 0.910	−0.044	(−0.11, 0.02) 0.184	0.080*	**(0.01, 0.14) 0.016**
TBW	−0.015	(−0.08, 0.05) 0.642	−0.057	(−0.13, 0.02) 0.086	0.084*	**(0.02, 0.15) 0.011**
TBW%	−0.080*	**(−0.14, −0.02) 0.015**	0.026	(−0.05, 0.09) 0.428	−0.131**	**(−0.19, −0.08)** < **0.001**
BM	0.000	(−0.07, 0.07) 0.992	−0.047	(−0.11, 0.02) 0.154	0.084*	**(0.02, 0.15) 0.011**
BMR	0.012	(−0.06, 0.08) 0.723	−0.053	(−0.12, 0.02) 0.108	0.085*	**(0.02, 0.15) 0.010**
VFR	0.032	(−0.04, 0.11) 0.333	−0.016	(−0.09, 0.06) 0.629	0.116**	**(0.04, 0.21)** < **0.001**
Wt.	0.033	(−0.04, 0.11) 0.318	−0.057	(−0.13, 0.02) 0.085	0.136**	**(0.07, 0.20)** < **0.001**
Ht.	−0.002	(−0.07, 0.06) 0.943	−0.015	(−0.08, 0.05) 0.643	0.029	(−0.04, 0.09) 0.381

*Note:* $ Correlation test. *p < 0.05, **p < 0.01 indicate statistical significance.

## 4 Discussion

In the present study, the relationship between DII, EAT, and body composition was investigated in a cross‐sectional study among university students. A total of 911 students were enrolled, of whom 49.5% were males and 50.5% were females with an age range between 18 and 30 years. In this study, we calculated DII and E‐DII from dietary data collected by FFQ, following correction of implausible energy and nutrient intake values, investigated eating attitude disorder by using the EAT‐26 questionnaire, and measured body composition.

The findings showed that most of the students (55.3%) had normal weight, with a mean DII score of −0.77 and mean EAT‐26 score of 15.07. Based on the DII score, the majority of students (75.4%) had an anti‐inflammatory dietary profile; however, when energy‐adjusted (E‐DII), this pattern reversed, with 96.8% of students classified as pro‐inflammatory, a divergence that reflects the substantial influence of total caloric intake on the crude DII in this population and is discussed further below.

In this study, 55.3% and 26.1% of students have normal weight and are overweight respectively with 76.1% non‐smokers. These results are in line with a previous study reported in the same university suggested that 56.7% and 24% of the students belong to normal and overweight categories, while 88.9% were non‐smokers [19]. Our study found that the diet of the students was anti‐inflammatory (mean DII = ‐0.77) as reported previously in Italian adults [20]. This indicates the inflammatory potential of the diet consumed by the study participants as revealed that the DII of 75.4% found among the students was anti‐inflammatory. This finding is consistent with a study among nutrition and dietetics students in Türkiye, in which 85.1% of participants were classified as having an anti‐inflammatory diet. Among these students, 70% had a normal BMI; however, no significant association was observed between DII scores and BMI (*p* = 0.378) [21]. Evidence from female university students in the UAE suggests a relationship between dietary inflammatory potential and mental health, as students in the highest E‐DII tertile had approximately threefold greater odds of anxiety and stress symptoms than those in the lowest tertile [22].

Consistent with these findings, greater dietary inflammatory potential was associated with poorer overall diet quality. In a cross‐sectional study of 4,365 Iranian adults, participants in the highest DII tertile had lower Healthy Eating Index (HEI) and Alternative Healthy Eating Index (AHEI) scores and lower adherence to a balanced dietary pattern [23]. Similarly, among college students, higher DII scores were positively correlated with dietary glycaemic index and inversely correlated with HEI‐2010 scores, indicating poorer overall diet quality [24].

Dietary inflammatory potential may be associated with body composition through pathways involving chronic low‐grade inflammation. Accordingly, we compared body‐composition and anthropometric measures between participants consuming pro‐inflammatory (positive DII score) and anti‐inflammatory (negative DII score) diets. No significant between‐group differences were observed in FM, MM, BMI, VFM, body weight, WC, or BM, nor in the distribution of BMI categories. These findings are partly consistent with a cross‐sectional study among Iranian women living with overweight or obesity, in which most unadjusted anthropometric measures did not differ between the lower‐ and higher‐DII groups [25]. However, after adjustment for potential confounders, the lower‐DII group had 1.5 kg more fat‐free mass and significantly lower fat mass than the higher‐DII group [25]. In contrast, a meta‐analysis of 22 studies, including cross‐sectional, case‐control, and cohort studies, found that participants in the highest DII categories had a higher BMI (mean difference: 0.81 kg/m^2^; 95% CI: 0.37–1.26) and greater odds of obesity (OR: 1.31; 95% CI: 1.14–1.50) than those in the lowest categories [26]. Similarly, a meta‐analysis of 32 observational studies, comprising 30 cross‐sectional and two cohort studies, reported that higher DII scores were associated with a 1.81‐cm greater waist circumference (95% CI: 0.79–2.84) [27]. Differences may reflect variations in participant characteristics, dietary assessment methods, DII calculation and classification, and the measurement of body composition.

In the present study, EAT‐26 scores were positively correlated with BMI, whereas DII scores were not significantly correlated with either BMI or EAT‐26 scores. Similarly, Çolak et al. reported no significant correlations between DII, BMI, and EAT‐40 scores among nutrition and dietetics students [21]. However, comparisons should consider that Çolak et al. used the original 40‐item EAT, whereas the present study used the shorter EAT‐26 [21].

Although DII scores were not significantly associated with BMI, fat mass, visceral fat rating, or body weight in our study, significant associations were observed with fat‐free mass and muscle mass. The association with fat‐free mass is consistent with findings among Iranian women living with overweight or obesity, in whom lower DII scores were associated with greater fat‐free mass after adjustment for potential confounders [25]. Similarly, a cross‐sectional study of 599 young adults (mean age: 20.41 years) found significant associations of DII scores with body weight and fat‐free mass after adjustment for age, sex, and total energy intake [28]. Although its fat‐free mass finding supports the present results, its finding for body weight differs from ours. Conversely, a study among university students in Tehran found no significant associations between DII scores and body weight, BMI, body fat, waist circumference, or visceral fat, which is consistent with our findings for these measures [29].

Evidence from older populations also supports a relationship between dietary inflammatory potential and musculoskeletal body composition. Among postmenopausal Korean women, higher DII scores were associated with greater odds of osteopenic obesity (OR = 2.76; 95% CI: 1.40–5.44) [30]. Furthermore, an analysis of older adults participating in NHANES found that higher E‐DII scores were associated with lower muscle mass and muscle strength and with greater odds of having both low muscle mass and low muscle strength [31].

Higher EAT‐26 scores were also significantly associated with several body‐composition indicators in the present study, including BMI, FM, MM, BM, body weight, BMR, and VFR. Consistent with these findings, a study of Iranian physical education students reported associations between disordered eating attitudes and BMI and indicators of central adiposity [32]. Similarly, a study of adolescent female ballet dancers and controls found that participants with EAT‐26 scores ≥ 20 had significantly higher body weight, BMI, waist and hip circumferences, fat mass, and fat‐free mass than those scoring < 20 [33].

### 4.1 Limitations and Strengths of the Study

The current study has some limitations that should be discussed. First, this was a cross‐sectional study; therefore, it was not possible to demonstrate a causal relationship between the variables studied. Second, the questionnaires that were used were self‐reported. So, there is a possibility that some participants reported eating habits and behaviors that did not actually describe their real habits. To ensure that the participants completed the questionnaires as effectively as possible, anonymity was ensured. Furthermore, in this study, no eating disorder diagnostic assessment was performed, though the EAT‐26 is a commonly used screening tool for disordered eating symptomatology. The third potential limitation is the lack of information on physical activity. Finally, the DII score was calculated using 28 of the 45 food parameters included in the original DII. This number falls within the commonly used range of 25–30 parameters and is considered sufficient to provide meaningful estimates of dietary inflammatory potential [34].

To our best knowledge, the present study is the first conducted in the UAE to explore the relationship between body composition, eating habits, and DII among university students. Moreover, the study included body composition measurements with BIA rather than traditional obesity indices, which make the results of the anthropometric characteristics more reliable than the self‐reported characteristics.

## 5 Conclusion

The present study findings suggest that, based on the corrected DII, most students (75.4%) had an anti‐inflammatory dietary profile, though this reversed under energy adjustment (E‐DII: 96.8% pro‐inflammatory), highlighting the importance of accounting for total caloric intake when interpreting dietary inflammatory indices in this population. Body fat percentage, fat mass, and total body water percentage differed significantly by DII group. The third of students (30.3%) reported major eating‐disorder risk and EAT‐26 score was significantly associated with a broad range of body‐composition measures, while DII showed only a weak association limited to total body water percentage. These findings suggest that eating attitudes may be a more consistent correlation of body composition than dietary inflammatory potential in this population.

## Author Contributions

**Leila Cheikh Ismail:** conceptualization, investigation, writing – original draft, writing – review and editing, supervision, methodology. **MoezAlIslam E. Faris:** methodology, conceptualization, writing – original draft, writing – review and editing, supervision, investigation. **Dana N. Abdelrahim:** formal analysis, writing – review and editing. **Maysm Shapsogh:** writing – review and editing, writing – original draft, methodology. **Amira Imad Al Mast:** investigation, writing – review and editing. **Hind Majdi Abuyounis:** investigation, writing – review and editing. **Sara Atheel Altekreeti:** investigation, writing – review and editing. **Lily Stojanovska:** writing – review and editing, methodology. **Ayesha S Al Dhaheri:** writing – review and editing, methodology.

## Funding

The authors have nothing to report.

## Conflicts of Interest

The authors declare no conflicts of interest.

## Transparency Statement

The lead author, Leila Cheikh Ismail, affirms that this manuscript is an honest, accurate, and transparent account of the study being reported; that no important aspects of the study have been omitted; and that any discrepancies from the study as planned have been explained.

## Supporting information


Supporting File

## Data Availability

The data that support the findings of this study are available from the corresponding author upon reasonable request.
