# Supplementary material for: Exploring the Relationship Between Dietary Inflammatory Index, Eating Disorder, and Body Composition Among University Students in the UAE: A Cross‐Sectional Study
Source: Health Sci Rep. 2026 Oct 8;9(10):e73357. doi: 10.1002/hsr2.73357 (PMC13650398; doi:10.1002/hsr2.73357)
Supplement: Supplementary file 1 [file HSR2-9-e73357-s001.docx]

**Supplementary Table S1: Sensitivity analysis - Comparison of body composition and EAT-26 score between E-DII anti-inflammatory and pro-inflammatory groups (n=911)***

| **Variable** | **Overall mean** | **Mean (SD)** | | **Mean Difference (95%CI)** | **P-value** |
| --- | --- | --- | --- | --- | --- |
|  |  | **Anti-inflammatory**  **(n=29)** | **Pro-inflammatory**  **(n=882)** |  |  |
| Weight (kg) | 70.06 (18.30) | 72.59 (17.85) | 69.97 (18.32) | 2.61 (-4.27, 9.50) | .444 |
| Height (cm) | 168.33 (9.91) | 167.24 (7.13) | 168.37 (9.99) | -1.13 (-3.91, 1.66) | .416 |
| Fat % | 22.41 (9.64) | 25.44 (9.74) | 22.31 (9.62) | 3.13 (-0.62, 6.89) | .099 |
| Fat Mass (kg) | 16.39 (10.30) | 19.14 (10.81) | 16.30 (10.28) | 2.84 (-1.32, 7.00) | .173 |
| FFM (kg) | 53.75 (13.10) | 53.45 (11.76) | 53.76 (13.15) | -0.32 (-4.87, 4.23) | .887 |
| Muscle Mass (kg) | 51.01 (12.44) | 50.76 (11.21) | 51.02 (12.48) | -0.26 (-4.59, 4.08) | .905 |
| TBW (L) | 38.06 (9.01) | 37.90 (8.47) | 38.06 (9.03) | -0.17 (-3.44, 3.11) | .918 |
| TBW % | 54.91 (6.50) | 52.52 (6.22) | 54.99 (6.50) | -2.48 (-4.88, -0.07) | .044* |
| Bone Mass (kg) | 2.71 (0.61) | 2.72 (0.55) | 2.71 (0.61) | 0.01 (-0.21, 0.22) | .936 |
| BMR (kcal) | 1,652.43 (374.08) | 1653.62 (334.88) | 1652.39 (375.47) | 1.23 (-128.30, 130.76) | .985 |
| VFR | 3.11 (3.42) | 3.76 (3.42) | 3.08 (3.42) | 0.68 (-0.64, 1.99) | .304 |
| BMI (kg/m2) | 24.51 (5.03) | 25.73 (4.98) | 24.47 (5.03) | 1.27 (-0.65, 3.19) | .187 |
| EAT-26 score | 15.07 (12.70) | 20.24 (15.96) | 14.90 (12.56) | 5.34 (0.65, 10.04) | .026* |

* Welch's t-test (independent samples, unequal variances). This table reports the same comparisons as Table 3, using the E-DII grouping in place of DII, as a sensitivity analysis discussed in the Results and Discussion. Note the small anti-inflammatory subgroup (n=29); estimates should be interpreted with caution and warrant replication. *P<0.05.
